# Di-μ-chlorido-bis­{chlorido[2-(2-furyl­methyl­imino­meth­yl)pyridine-κ^2^ *N*,*N*′]nickel(II)}

**DOI:** 10.1107/S1600536808015183

**Published:** 2008-05-24

**Authors:** Dong-Sheng Xia, Wu Chen, Xin-Long Tang, Qing-Fu Zeng

**Affiliations:** a Engineering Research Center for Clean Production of Textile Printing, Ministry of Education, Wuhan University of Science & Engineering, Wuhan 430073, People’s Republic of China

## Abstract

The title dinuclear nickel(II) complex, [Ni_2_Cl_4_(C_11_H_10_N_2_O)_2_], lies on a centre of symmetry located at the centroid of the four-membered ring formed by the two Ni atoms and the bridging chloride ions. The Ni^II^ atom is five-coordinated in a square-pyramidal geometry by the imine and pyridine N atoms of the Schiff base ligand, and by one terminal and two bridging Cl atoms. The Ni⋯Ni distance is 3.506 (2) Å. The O atom of the furan substituent in the ligand unit is not involved in coordination to the Ni atom.

## Related literature

For related structures, see: Cheng *et al.* (2007 ▶); Li *et al.* (2007 ▶); Qiu *et al.* (2006 ▶); Shi *et al.* (2007 ▶); Wang *et al.* (2005 ▶); Zhu *et al.* (2003 ▶).
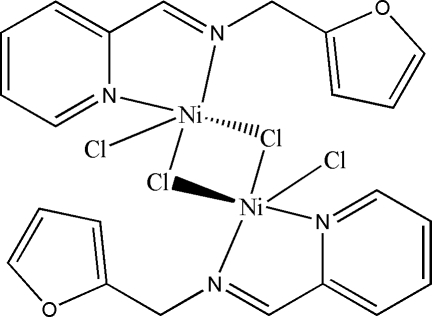


## Experimental

### 

#### Crystal data

[Ni_2_Cl_4_(C_11_H_10_N_2_O)_2_]
*M* *_r_* = 631.64
Triclinic, 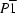

*a* = 8.0439 (8) Å
*b* = 8.5659 (8) Å
*c* = 10.0610 (9) Å
α = 77.522 (8)°
β = 72.040 (7)°
γ = 70.132 (8)°
*V* = 615.39 (10) Å^3^
*Z* = 1
Mo *K*α radiation
μ = 1.99 mm^−1^
*T* = 298 (2) K
0.30 × 0.30 × 0.28 mm

#### Data collection

Bruker SMART CCD area-detector diffractometer
Absorption correction: multi-scan (*SADABS*; Sheldrick, 1996 ▶) *T*_min_ = 0.554, *T*_max_ = 0.572
2585 measured reflections
2408 independent reflections
1971 reflections with *I* > 2σ(*I*)
*R*_int_ = 0.020

#### Refinement

*R*[*F* ^2^ > 2σ(*F* ^2^)] = 0.040
*wR*(*F* ^2^) = 0.099
*S* = 1.07
2408 reflections
154 parameters
H-atom parameters constrained
Δρ_max_ = 0.51 e Å^−3^
Δρ_min_ = −0.58 e Å^−3^

### 

Data collection: *SMART* (Bruker, 1998 ▶); cell refinement: *SAINT* (Bruker, 1998 ▶); data reduction: *SAINT*; program(s) used to solve structure: *SHELXS97* (Sheldrick, 2008 ▶); program(s) used to refine structure: *SHELXL97* (Sheldrick, 2008 ▶); molecular graphics: *SHELXTL* (Sheldrick, 2008 ▶); software used to prepare material for publication: *SHELXTL*.

## Supplementary Material

Crystal structure: contains datablocks global, I. DOI: 10.1107/S1600536808015183/sj2503sup1.cif

Structure factors: contains datablocks I. DOI: 10.1107/S1600536808015183/sj2503Isup2.hkl

Additional supplementary materials:  crystallographic information; 3D view; checkCIF report

## Figures and Tables

**Fig. 1.**
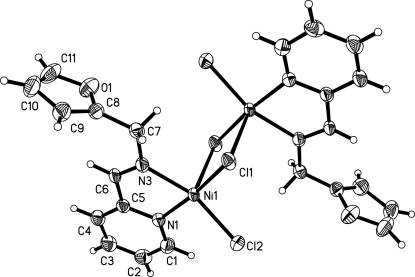
The structure of (I) showing 30% probability displacement ellipsoids and the atom-numbering scheme. Numbered atoms are related to un-numbered atoms by the symmetry code 1-x, 2-y, 1-z.

## supplementary crystallographic information

### Comment

As part of our ongoing interest in the structure of nickel(II) complexes (Zhu *et al.*, 2003), we report herein the crystal structure of the title compound, a new centrosymmetric dinuclear nickel(II) complex, (I), Fig. 1, derived from the Schiff base ligand furan-2-ylmethyl-(1-pyridin-2-ylmethylidene)amine.

The Ni^II^ atom in (I) is five-coordinate in a square pyramidal geometry, binding to the imine and pyridine N atoms of the Schiff base ligand, and to one terminal Cl and two bridging Cl atoms. The Ni···Ni distance is 3.506 (2) Å. The dihedral angle between the benzene ring and the furan ring is 73.3 (3) °. The O atom of the furan substituent in the ligand lies well away from the coordination sphere of the Ni atom.  The coordinate bond values (Table 1) are comparable to values observed in other similar nickel(II) complexes (Shi *et al.*, 2007; Li *et al.*, 2007; Cheng *et al.*, 2007; Qiu *et al.*, 2006; Wang *et al.*, 2005).

### Experimental

Pyridine-2-carbaldehyde (10.7 mg, 0.1 mmol), furan-2-ylmethylamine (9.7 mg, 0.1 mmol), and NiCl_2_.6H_2_O (23.8 mg, 0.1 mmol) were dissolved in methanol (30 ml). The mixture was stirred for 30 min at room temperature. The resulting solution was left in air for a few days, yielding green crystals.

### Refinement

H atoms were placed in idealized positions and constrained to ride on their parent atoms with C–H distances in the range 0.93–0.97 Å, and with *U*_iso_(H) set at 1.2*U*_eq_(C).

### Crystal data

**Table:**

[Ni_2_Cl_4_(C_11_H_10_N_2_O)_2_]	*Z* = 1
*M**_r_* = 631.64	*F*_000_ = 320
Triclinic, *P*1	*D*_x_ = 1.704 Mg m^−^^3^
Hall symbol: -P 1	Mo *K*α radiation λ = 0.71073 Å
*a* = 8.0439 (8) Å	Cell parameters from 1237 reflections
*b* = 8.5659 (8) Å	θ = 2.4–25.3º
*c* = 10.0610 (9) Å	µ = 1.99 mm^−^^1^
α = 77.522 (8)º	*T* = 298 (2) K
β = 72.040 (7)º	Block, green
γ = 70.132 (8)º	0.30 × 0.30 × 0.28 mm
*V* = 615.39 (10) Å^3^	

### Data collection

**Table:**

Bruker SMART CCD area-detector diffractometer	2408 independent reflections
Radiation source: fine-focus sealed tube	1971 reflections with *I* > 2σ(*I*)
Monochromator: graphite	*R*_int_ = 0.020
*T* = 298(2) K	θ_max_ = 26.0º
ω scans	θ_min_ = 2.6º
Absorption correction: multi-scan(SADABS; Sheldrick, 1996)	*h* = 0→9
*T*_min_ = 0.554, *T*_max_ = 0.572	*k* = −9→10
2585 measured reflections	*l* = −11→12

### Refinement

**Table:**

Refinement on *F*^2^	Secondary atom site location: difference Fourier map
Least-squares matrix: full	Hydrogen site location: inferred from neighbouring sites
*R*[*F*^2^ > 2σ(*F*^2^)] = 0.040	H-atom parameters constrained
*wR*(*F*^2^) = 0.099	*w* = 1/[σ^2^(*F*_o_^2^) + (0.0486*P*)^2^ + 0.2142*P*] where *P* = (*F*_o_^2^ + 2*F*_c_^2^)/3
*S* = 1.07	(Δ/σ)_max_ < 0.001
2408 reflections	Δρ_max_ = 0.51 e Å^−^^3^
154 parameters	Δρ_min_ = −0.58 e Å^−^^3^
Primary atom site location: structure-invariant direct methods	Extinction correction: none

### Special details

**Table:**

Geometry. All e.s.d.'s (except the e.s.d. in the dihedral angle between two l.s. planes) are estimated using the full covariance matrix. The cell e.s.d.'s are taken into account individually in the estimation of e.s.d.'s in distances, angles and torsion angles; correlations between e.s.d.'s in cell parameters are only used when they are defined by crystal symmetry. An approximate (isotropic) treatment of cell e.s.d.'s is used for estimating e.s.d.'s involving l.s. planes.
Refinement. Refinement of *F*^2^ against ALL reflections. The weighted *R*-factor *wR* and goodness of fit *S* are based on *F*^2^, conventional *R*-factors *R* are based on *F*, with *F* set to zero for negative *F*^2^. The threshold expression of *F*^2^ > σ(*F*^2^) is used only for calculating *R*-factors(gt) *etc*. and is not relevant to the choice of reflections for refinement. *R*-factors based on *F*^2^ are statistically about twice as large as those based on *F*, and *R*- factors based on ALL data will be even larger.

### Fractional atomic coordinates and isotropic or equivalent isotropic displacement parameters (Å^2^)

**Table:**

	*x*	*y*	*z*	*U*_iso_*/*U*_eq_	
Ni1	0.72650 (5)	1.00555 (5)	0.45738 (4)	0.03767 (16)	
Cl1	0.46003 (12)	1.19011 (10)	0.55654 (10)	0.0481 (2)	
Cl2	0.76194 (14)	1.16826 (13)	0.24879 (11)	0.0608 (3)	
O1	0.6666 (5)	0.6517 (4)	0.9351 (3)	0.0729 (9)	
N1	0.9716 (4)	0.8340 (4)	0.3931 (3)	0.0444 (7)	
C11	0.7243 (7)	0.5754 (6)	1.0541 (5)	0.0848 (16)	
H11	0.7256	0.4670	1.0955	0.102*	
N3	0.7676 (4)	0.8807 (3)	0.6475 (3)	0.0418 (7)	
C1	1.0714 (5)	0.8105 (5)	0.2615 (4)	0.0576 (10)	
H1	1.0313	0.8837	0.1871	0.069*	
C2	1.2312 (6)	0.6821 (5)	0.2320 (5)	0.0617 (11)	
H2	1.2962	0.6683	0.1392	0.074*	
C3	1.2941 (5)	0.5746 (5)	0.3406 (5)	0.0608 (11)	
H3	1.4016	0.4870	0.3227	0.073*	
C4	1.1937 (5)	0.5998 (5)	0.4771 (4)	0.0533 (10)	
H4	1.2343	0.5305	0.5528	0.064*	
C5	1.0333 (5)	0.7283 (4)	0.4998 (4)	0.0429 (8)	
C6	0.9147 (4)	0.7611 (4)	0.6387 (4)	0.0428 (8)	
H6	0.9458	0.6960	0.7193	0.051*	
C7	0.6364 (5)	0.9170 (5)	0.7850 (4)	0.0477 (9)	
H7A	0.6191	1.0315	0.7966	0.057*	
H7B	0.5196	0.9099	0.7827	0.057*	
C8	0.6882 (5)	0.8072 (5)	0.9094 (4)	0.0479 (9)	
C9	0.7552 (6)	0.8277 (6)	1.0092 (4)	0.0658 (11)	
H9	0.7815	0.9228	1.0160	0.079*	
C10	0.7779 (7)	0.6769 (7)	1.1019 (5)	0.0820 (16)	
H10	0.8223	0.6537	1.1813	0.098*	

### Atomic displacement parameters (Å^2^)

**Table:**

	*U*^11^	*U*^22^	*U*^33^	*U*^12^	*U*^13^	*U*^23^
Ni1	0.0292 (2)	0.0323 (2)	0.0470 (3)	−0.00427 (17)	−0.01482 (18)	0.00379 (17)
Cl1	0.0407 (5)	0.0327 (4)	0.0685 (6)	−0.0016 (4)	−0.0209 (4)	−0.0061 (4)
Cl2	0.0525 (6)	0.0567 (6)	0.0665 (6)	−0.0179 (5)	−0.0227 (5)	0.0201 (5)
O1	0.094 (2)	0.0557 (18)	0.0644 (19)	−0.0237 (17)	−0.0199 (17)	0.0038 (14)
N1	0.0373 (16)	0.0441 (16)	0.0497 (17)	−0.0115 (13)	−0.0144 (13)	0.0024 (13)
C11	0.093 (4)	0.062 (3)	0.058 (3)	0.002 (3)	−0.005 (3)	0.015 (2)
N3	0.0380 (15)	0.0382 (15)	0.0498 (17)	−0.0091 (13)	−0.0171 (13)	−0.0013 (13)
C1	0.052 (2)	0.061 (2)	0.052 (2)	−0.010 (2)	−0.0134 (19)	0.0004 (19)
C2	0.050 (2)	0.063 (3)	0.062 (3)	−0.011 (2)	0.000 (2)	−0.017 (2)
C3	0.044 (2)	0.048 (2)	0.078 (3)	−0.0007 (18)	−0.012 (2)	−0.010 (2)
C4	0.041 (2)	0.043 (2)	0.067 (3)	−0.0028 (16)	−0.0165 (19)	−0.0015 (18)
C5	0.0357 (18)	0.0364 (18)	0.055 (2)	−0.0091 (14)	−0.0146 (16)	−0.0008 (15)
C6	0.0351 (18)	0.0396 (18)	0.052 (2)	−0.0077 (15)	−0.0197 (16)	0.0043 (15)
C7	0.0371 (19)	0.045 (2)	0.054 (2)	−0.0053 (16)	−0.0105 (16)	−0.0041 (16)
C8	0.042 (2)	0.045 (2)	0.048 (2)	−0.0045 (16)	−0.0093 (16)	−0.0051 (16)
C9	0.066 (3)	0.076 (3)	0.054 (2)	−0.019 (2)	−0.016 (2)	−0.009 (2)
C10	0.074 (3)	0.099 (4)	0.049 (3)	0.000 (3)	−0.019 (2)	0.004 (3)

### Geometric parameters (Å, °)

**Table:**

Ni1—N1	2.030 (3)	C2—C3	1.374 (6)
Ni1—N3	2.044 (3)	C2—H2	0.9300
Ni1—Cl2	2.2506 (10)	C3—C4	1.382 (5)
Ni1—Cl1	2.2690 (10)	C3—H3	0.9300
Ni1—Cl1^i^	2.6496 (10)	C4—C5	1.375 (5)
Cl1—Ni1^i^	2.6496 (10)	C4—H4	0.9300
O1—C8	1.361 (5)	C5—C6	1.452 (5)
O1—C11	1.369 (5)	C6—H6	0.9300
N1—C1	1.337 (5)	C7—C8	1.473 (5)
N1—C5	1.350 (4)	C7—H7A	0.9700
C11—C10	1.317 (7)	C7—H7B	0.9700
C11—H11	0.9300	C8—C9	1.343 (5)
N3—C6	1.268 (4)	C9—C10	1.412 (6)
N3—C7	1.479 (4)	C9—H9	0.9300
C1—C2	1.377 (5)	C10—H10	0.9300
C1—H1	0.9300		
			
N1—Ni1—N3	79.68 (11)	C2—C3—C4	118.4 (4)
N1—Ni1—Cl2	92.50 (9)	C2—C3—H3	120.8
N3—Ni1—Cl2	160.74 (8)	C4—C3—H3	120.8
N1—Ni1—Cl1	172.70 (9)	C5—C4—C3	119.3 (4)
N3—Ni1—Cl1	93.38 (8)	C5—C4—H4	120.4
Cl2—Ni1—Cl1	93.42 (4)	C3—C4—H4	120.4
N1—Ni1—Cl1^i^	93.04 (8)	N1—C5—C4	122.3 (3)
N3—Ni1—Cl1^i^	91.84 (8)	N1—C5—C6	114.2 (3)
Cl2—Ni1—Cl1^i^	106.23 (4)	C4—C5—C6	123.5 (3)
Cl1—Ni1—Cl1^i^	89.40 (3)	N3—C6—C5	118.3 (3)
Ni1—Cl1—Ni1^i^	90.60 (3)	N3—C6—H6	120.8
C8—O1—C11	106.2 (4)	C5—C6—H6	120.8
C1—N1—C5	118.0 (3)	C8—C7—N3	116.0 (3)
C1—N1—Ni1	128.3 (3)	C8—C7—H7A	108.3
C5—N1—Ni1	113.7 (2)	N3—C7—H7A	108.3
C10—C11—O1	110.6 (4)	C8—C7—H7B	108.3
C10—C11—H11	124.7	N3—C7—H7B	108.3
O1—C11—H11	124.7	H7A—C7—H7B	107.4
C6—N3—C7	121.3 (3)	C9—C8—O1	109.5 (4)
C6—N3—Ni1	114.1 (2)	C9—C8—C7	133.0 (4)
C7—N3—Ni1	124.5 (2)	O1—C8—C7	117.5 (3)
N1—C1—C2	122.4 (4)	C8—C9—C10	106.9 (5)
N1—C1—H1	118.8	C8—C9—H9	126.6
C2—C1—H1	118.8	C10—C9—H9	126.6
C3—C2—C1	119.6 (4)	C11—C10—C9	106.8 (4)
C3—C2—H2	120.2	C11—C10—H10	126.6
C1—C2—H2	120.2	C9—C10—H10	126.6
			
N1—Ni1—Cl1—Ni1^i^	−109.6 (7)	N1—C1—C2—C3	−1.1 (7)
N3—Ni1—Cl1—Ni1^i^	−91.81 (8)	C1—C2—C3—C4	−0.2 (6)
Cl2—Ni1—Cl1—Ni1^i^	106.22 (4)	C2—C3—C4—C5	1.4 (6)
Cl1^i^—Ni1—Cl1—Ni1^i^	0.0	C1—N1—C5—C4	0.2 (5)
N3—Ni1—N1—C1	178.4 (3)	Ni1—N1—C5—C4	177.7 (3)
Cl2—Ni1—N1—C1	−19.4 (3)	C1—N1—C5—C6	−178.9 (3)
Cl1—Ni1—N1—C1	−163.6 (5)	Ni1—N1—C5—C6	−1.4 (4)
Cl1^i^—Ni1—N1—C1	87.0 (3)	C3—C4—C5—N1	−1.5 (6)
N3—Ni1—N1—C5	1.3 (2)	C3—C4—C5—C6	177.5 (4)
Cl2—Ni1—N1—C5	163.5 (2)	C7—N3—C6—C5	177.4 (3)
Cl1—Ni1—N1—C5	19.3 (8)	Ni1—N3—C6—C5	0.4 (4)
Cl1^i^—Ni1—N1—C5	−90.1 (2)	N1—C5—C6—N3	0.7 (5)
C8—O1—C11—C10	0.5 (5)	C4—C5—C6—N3	−178.4 (3)
N1—Ni1—N3—C6	−0.9 (2)	C6—N3—C7—C8	0.5 (5)
Cl2—Ni1—N3—C6	−68.1 (4)	Ni1—N3—C7—C8	177.2 (2)
Cl1—Ni1—N3—C6	−178.6 (2)	C11—O1—C8—C9	−0.6 (5)
Cl1^i^—Ni1—N3—C6	91.9 (2)	C11—O1—C8—C7	179.7 (3)
N1—Ni1—N3—C7	−177.8 (3)	N3—C7—C8—C9	103.2 (5)
Cl2—Ni1—N3—C7	114.9 (3)	N3—C7—C8—O1	−77.1 (4)
Cl1—Ni1—N3—C7	4.4 (3)	O1—C8—C9—C10	0.4 (5)
Cl1^i^—Ni1—N3—C7	−85.1 (3)	C7—C8—C9—C10	−179.9 (4)
C5—N1—C1—C2	1.0 (6)	O1—C11—C10—C9	−0.3 (6)
Ni1—N1—C1—C2	−175.9 (3)	C8—C9—C10—C11	−0.1 (6)

Symmetry codes: (i) −*x*+1, −*y*+2, −*z*+1.
